# Predictors of the therapeutic effect of sivelestat in patients with acute lung injury associated with systemic inflammatory response syndrome

**DOI:** 10.1186/s40780-016-0051-x

**Published:** 2016-08-24

**Authors:** Takuma Ozawa, Kiyoshi Mihara, Nobuhiro Yasuno

**Affiliations:** 1 Department of Pharmacy, Kan-etsu Hospital, 145-1 Suneori, Tsuruashima-shi, Saitama, 350-2213 Japan; 2 Research Center for Clinical Pharmacy, Faculty of Pharmacy, Musashino University, 1-1-20 Shinmachi, Nishitokyo-shi, Tokyo, 202-8585 Japan

**Keywords:** Sivelestat, Acute lung injury, Efficacy prediction, Logistic regression analysis, Red blood cell

## Abstract

**Background:**

Sivelestat, a neutrophil elastase inhibitor, was previously approved in Japan for the treatment of acute lung injury associated with systemic inflammatory response syndrome. However, sivelestat produced inconsistent therapeutic benefits. This study aimed to identify factors predicting the therapeutic effects of sivelestat.

**Methods:**

We enrolled 53 mechanically ventilated patients who received sivelestat. The patients were classified as effective (*n* = 28) if they were weaned from the ventilator within 28 days, or as ineffective groups (*n* = 25). Patient characteristics were compared between these groups and multivariate logistic regression analysis was used to identify predictive factors. A validation study was then conducted in sivelestat-free patients.

**Results:**

A high red blood cell count and low hydrogen ion concentration were significantly associated with a higher ventilator weaning rate in patients receiving sivelestat. The validation study revealed that the hydrogen ion concentration value also significantly associated with ventilator weaning in patients who did not receive sivelestat.

**Conclusions:**

Although hydrogen ion concentration was inversely associated with the ventilator weaning rate, it did not predict sivelestat efficacy. This study indicated that acute lung injury patients with a high red blood cell count would derive the most benefit from sivelestat administration.

## Background

Acute lung injury (ALI) is a complex disorder caused by inflammation in response to conditions such as pneumonia and sepsis. ALI is characterized by a neutrophilic inflammatory response, associated with increased pulmonary vascular permeability [1]. Neutrophil elastase (NE) is one of the proteases released from the neutrophils that accumulate in the lung [2]. It degrades lung connective tissues and enhances lung vasopermeability, resulting in ALI. Furthermore, NE increases the production of neutrophil migration factor and is recognized as an important driver of systemic inflammatory response syndrome (SIRS) [3].

Sivelestat is a selective NE inhibitor, which binds directly to NE and inactivates it by a reversible acylation-deacylation mechanism [4–6]. In addition, it relaxes vascular smooth muscle [7]. Sivelestat was approved in Japan as a therapeutic drug for ALI associated with SIRS, but is also used in other situations. For example, perioperative administration of sivelestat for esophagectomy improves postoperative respiratory function and shortens the duration of hospitalization and of mechanical ventilation [8–12]. Moreover, sivelestat is administered for pediatric cardiopulmonary bypass surgery because it attenuates the perioperative inflammatory response [13]. Sivelestat also shortens the duration of mechanical ventilation, intensive care unit stay, and hospitalization due to severe respiratory failure after thoracic aortic surgery [14].

In a post-marketing clinical trial (phase IV) conducted in Japan [4], the adjusted mean number of ventilator-free days (VFD) was significantly higher in the sivelestat group than in the control group (*P* = 0.0022). However, the Sivelestat Trial in ALI Patients Requiring Mechanical Ventilation (STRIVE) study [15] did not identify a significant increase in VFDs in the sivelestat group, as compared with the control group, and the final analysis revealed no effect of sivelestat on VFD (day 1–28). Furthermore, the 180-day all-cause mortality was higher in the sivelestat group than in the control group (*P* = 0.006). This resulted in the discontinuation of this clinical trial and sivelestat thus failed to receive approval in other countries, such as the USA.

These trials [4, 15] found that the efficacy of sivelestat was inconsistent and it would therefore be beneficial to identify the factors determining the differential effects of sivelestat in patients who respond to treatment, versus the non-responders. The purpose of this case-control study was to identify factors predicting the therapeutic effects of sivelestat.

## Methods

### Patients

The case-control study was conducted in compliance with the Strengthening the Reporting of Observational Studies in Epidemiology statement and the Declaration of Helsinki. The protocol (H024-003) was approved by the institutional review board at Kan-etsu hospital (Saitama, Japan). Fifty-three patients on mechanical ventilation, who were administered sivelestat in the hospital from April 2006 to March 2012, were enrolled in this study.

### Study design

The patients were divided into two groups, according to whether they were categorized as effective or ineffective. Patients in the effective group were weaned from the ventilator within 28 days. VFDs (the primary endpoint of the phase IV study in Japan [14]) and all-cause mortality (the primary endpoint of the STRIVE study [15]) were both assessed within 28 days in these studies; we therefore used a 28-day monitoring period in the present study.

Data from the day before sivelestat administration were used for this analysis. If these were unavailable, we used the data from two days before sivelestat administration or from the day of sivelestat administration. Vital signs and SpO_2_ were not included in the analysis because they were affected by concomitant drugs or ventilation. We initially compared the effective and ineffection group data. We subsequently identified predictive factors using the multivariate analysis described below.

### Validation

The fators identified using the approach described above may predict the patient response to sivelestat or may represent independent prognostic indicators. To investigate this, we conducted a validation study in the absence of sivelestat. Patients who were using a medical ventilator but were not administered sivelestat were enrolled in Kan-etsu hospital from January 2013 to December 2014. We excluded perioperative patients and those who had been hospitalized for < 24 h from this study.

Patients were divided into two groups, depending on whether they were weaned from the ventilator within 28 days (the weaning group) or not (the no weaning group).

These groups were compared using the approach described in Study design.

### Statistics

We used statistical analyses to investigate which factors predicted the therapeutic effect of sivelestat. We performed an *F*-test to determine whether each factor exhibited a homoscedastic distribution. A *t*-test was used in the initial screening of the effective and ineffective groups if the distribution showed homoscedasticity; if not, Welch’s *t*-test was used. The numbers of male patients, sepsis, and DIC in the two groups were analyzed using the *Chi*-square test. We then performed multivariate logistic regression analysis (stepwise: likelihood ratio) using SPSS® (SPSS Inc., Chicago, USA) to identify factors that showed a significant association with a high mechanical ventilation weaning rate. We employed the stepwise method because the method is suitable to control confounding factors in multivariate logistic regression analysis.

## Results

### Case-control study

The profiles of the effective (*n* = 28) and ineffective (*n* = 25) patient groups are shown in Table 1. The age, hydrogen ion concentration ([H^+^]), and PCO_2_ values were significantly lower in the effective group than in the ineffective group. In contrast, the red blood cell count (RBC) and PO_2_ were significantly higher in the effective group than in the ineffective group. Other factors showed no statistically significant differences between these groups.

**Table 1:** Profiles of the patients included in the case-control study

	Effective group (*n* = 28)	Ineffective group (*n* = 25)	*P*
Age(yr)	67.7 ± 14.5	77.0 ± 8.4	0.006 ^b^
Male (%)	21 (75.0 %)	20 (80.0 %)	0.664 ^c^
Sepsis (%)	8 (28.6 %)	13 (52.0 %)	0.082 ^c^
DIC (%)	9 (32.1 %)	11 (44.0 %)	0.374 ^c^
ALT(IU/L)	46.8 ± 38.9	29.3 ± 26.4	0.064 ^a^
AST(IU/L)	67.3 ± 61.9	75.1 ± 119.1	0.772 ^b^
LDH(IU/L)	569 ± 222	670 ± 744	0.350 ^b^
ALP(IU/L)	306 ± 160	500 ± 829	0.315 ^b^
γ-GTP(IU/L)	68.9 ± 80.2	40.2 ± 34.6	0.121 ^b^
BUN(mg/dL)	31.9 ± 20.8	37.3 ± 21.6	0.353 ^a^
Scr(mg/dL)	2.0 ± 2.7	2.1 ± 2.6	0.961 ^a^
RBC(×10^4^/μL)	376.1 ± 84.9	327.2 ± 72.1	0.029 ^a^
WBC(/μL)	11,121 ± 7299	13,441 ± 8549	0.292 ^a^
Neut(%)	88.4 ± 9.3	85.9 ± 10.3	0.607 ^a^
Plt(×10^4^/μL)	18.8 ± 9.8	16.0 ± 9.8	0.295 ^a^
Alb(g/dL)	3.1 ± 2.0	2.4 ± 0.6	0.127 ^b^
CRP(mg/dL)	19.6 ± 11.2	15.5 ± 9.4	0.168 ^a^
[H^+^](×10^−8^)	3.639 ± 0.667	4.422 ± 0.936	0.001 ^b^
PO_2_(mmHg)	93.2 ± 48.1	61.4 ± 24.7	0.004 ^b^
PCO_2_(mmHg)	36.6 ± 9.3	47.7 ± 17.5	0.007 ^b^
Dose(mg/kg/h)	0.216 ± 0.049	0.209 ± 0.046	0.785 ^a^

The data were expressed as the mean ± standard deviation, except for the number of males, sepsis, and DIC. The value of [H^+^] was calculated as follows: [H^+^] = 10^–pH^

a: Student *t*-test, b: Welch’s *t*-test, c: *Chi*-square test

**Fig. 1:**
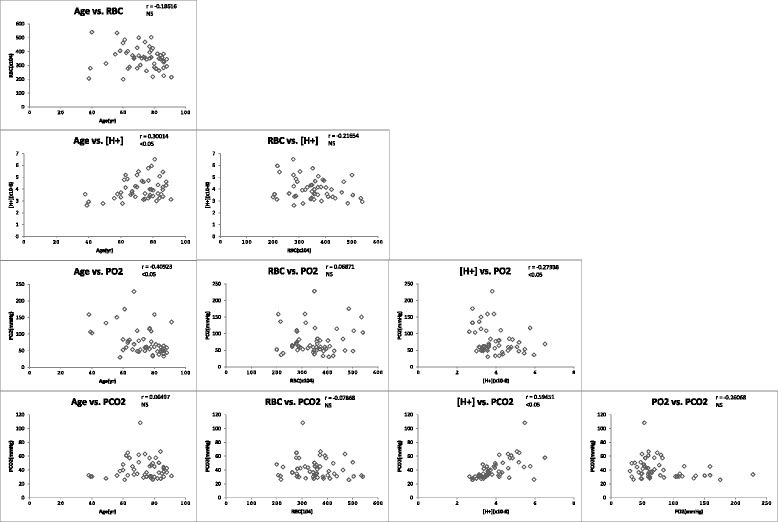
The relationship between factors adopted in the main analysis. RBC: red blood cell, [H+]: hydrogen ion concentration, PO2: oxygen partial pressure, PCO2: carbon dioxide partial pressure

We used the factors that differed significantly between these study groups (age, RBC, [H^+^], PO_2_, and PCO_2_) to perform multivariate logistic regression analysis. The relationships between these factors are shown in Fig. 1. The value of PO_2_ slightly correlated with that of [H^+^]. The results of the multivariate logistic regression analysis are shown in Table 2.

**Table 2:** Logistic regression analysis

	B	S.E.	*p*-value	AOR	95 % CI
RBC	0.012	0.006	0.044	1.012	1.000–1.024
[H^+^]	−1.194	0.510	0.019	0.303	0.112–0.824
PO_2_	0.030	0.014	0.031	1.030	1.003–1.058
constant	−1.412	2.856	0.621		

*B* regression coefficient

*S.E.* standard error

*AOR* adjusted odds ratio

*95 % CI* 95 % confidence interval

Age and PCO_2_, which correlated with [H^+^] and/or PO_2_, were dropped from the process. Three factors (RBC, [H^+^], and PO_2_) were therefore identified as candidates for the prediction of the therapeutic effect of sivelestat.

### Validation

The validation study included 105 patients receiving medical ventilation in the absence of sivelestat in Kan-etsu hospital from January 2013 to December 2014. The patient’s age, sex, RBC, and [H^+^] values in these weaning and no weaning groups are shown in Table 3. The [H^+^] value was significantly higher in the no weaning ventilator group than in the weaning group (*P* = 0.006) and there was no significant difference in RBC between these groups (*P* = 0.13).

**Table 3:** Profiles of the patients included in the validation study

		Weaning ventilator group (*n* = 56)	Non weaning ventilator group (*n* = 49)	*P*
Basical data	Age(yr)	77.5 ± 14.0	79.1 ± 9.8	0.521 ^b^
	Male (%)	29 (51.8 %)	35 (71.4 %)	0.040 ^c^
Candidates	RBC(×10^4^/μL)	385.6 ± 72.9	361.7 ± 91.2	0.139 ^a^
	[H^+^](×10^−8^)	4.661 ± 1.534	6.158 ± 3.418	0.006 ^b^

The data were expressed as the mean ± standard deviation, except for the number of males

The value of [H^+^] was calculated as follows: [H^+^] = 10^–pH^

a: Student *t*-test, b: Welch’s *t*-test, c: *Chi*-square test

## Discussion

Although the efficacy and safety of Sivelestat in the Japanese patients was demonstrated in the phase III double-blind study and the postparket study, the difference in the 28-day ventilator-weaning rate was only 13 % between the sivelestat and control group in the postparket study and the negative results was reported in the STRIVE study [4, 15, 16]. The reason why the efficacy was small or negative would be the presence of the responder and non-responder. Thus, we attempted to identify factors predicting the therapeutic effects of sivelestat. This case-control study is the first trial to find predictors of the therapeutic effect of sivelestat in patients with ALI associated with SIRS.

We initially evaluated the differences between the effective (ventilator weaning) and ineffective groups of patients who were administered with sivelestat. This logistic regression analysis revealed that three factors, [H^+^], RBC, and PO_2_, were significantly associated with the mechanical ventilation weaning rate of these patients.

A predictive formula was developed:

1 $$ \ln \left(\mathrm{y}/1 - \mathrm{y}\right) = 0.012 \times \mathrm{R}\mathrm{B}\mathrm{C} - 1.194 \times \left[{\mathrm{H}}^{+}\right] + 0.030 \times \mathrm{P}{\mathrm{O}}_2 - 1.412 $$

where y was the probability of a good response to sivelestat. According to this formula, a higher RBC, lower [H^+^], and higher PO_2_ were associated with a greater probability of a good therapeutic response to sivelestat.

Formula 1 allows calculation of the probability of sivelestat effectiveness in a patient using the values of these predictive factors. The normal reference values for [H^+^], RBC, and PO_2_ are 3.548 × 10^−8^ (corresponding to a pH of 7.45), 450 (× 10^4^/μL), and 100, respectively. Substitution of these reference values into formula 1 produces a probability of 94 %. If one of these factors ([H^+^], RBC, or PO_2_) changed to 5.853 × 10^−8^ (pH = 7.23), 221, or 8.3, respectively, the probability was reduced to 50 %.

This indicated that PO_2_ was unlikely to modulate the patient response to sivelestat significantly, because a PO_2_ value of 8.3 is not realistic in the clinical situation. On the other hand, [H^+^] and RBC may have greater effect on sivelestat response because a [H^+^] value of 5.853 × 10^−8^ (pH = 7.23), or a RBC value of 221 are often observed in hospitalized patients.

Respiratory failure induces severe acidosis, where pulmonary CO_2_ elimination decreases (respiratory acidosis) and/or an oxygen deficit (lactic acidosis) occurs. Our findings suggested that as respiratory function declined, sivelestat became less effective. Red blood cells transport oxygen within the body and a reduced RBC therefore indicates oxygen deficiency, which necessitates a longer ventilation period.

Although we found two candidate predictive factors in the first study, it was possible that these predicted ventilator weaning, regardless of sivelestat administration. Thus, we conducted a validation study of [H^+^] and RBC values in patients who did not receive sivelestat.

The mean [H+] value was significantly higher in the no weaning group, as compared with the weaning group, indicating that this parameter predicted ventilator weaning, even in the absence of sivelestat. On the other hand, RBC showed little effect on the weaning rate of these patients, indicating that only RBC predicted the therapeutic effect of sivelestat.

Patients who received dialysis therapy were expected to have variable RBC values. Then, we count number of dialyzing patients in the case-control study. The numbers of dialyzing patients were 8 in the effective group and 6 in the ineffective group. The ratio of the dialyzing patients was not significantly different between the groups (*chi*-square test, *p* = 0.706).

Because a few patients were administered sivelestat in Kan-etsu hospital, the patient numbers of effective and ineffective group were 28 and 25, respectively. Such small sample size is limitation of this study. The study of large sample size is needed.

## Conclusion

Evaluated [H^+^], RBC and PO_2_ levels prior to sivelestat administration predicted that it would produce less therapeutic benefit. The RBC represented a particularly valuable predictor of this therapeutic effect. ALI patients with high RBC levels may be the best candidates for sivelestat administration.

## Abbreviations

ALI, acute lung injury; NE, neutrophil elastase; SIRS, systemic inflammatory response syndrome; STRIVE, Sivelestat Trial in ALI Patients Requiring Mechanical Ventilation; VFD, ventilator-free days
